# Opioid System Modulates the Immune Function: A Review

**Published:** 2016

**Authors:** Xuan Liang, Renyu Liu, Chunhua Chen, Fang Ji, Tianzuo Li

**Affiliations:** 1 Department of Anesthesiology and Critical Care, Perelman School of Medicine at the University of Pennsylvania; 2 Department of Anesthesiology, Tongren Hospital, Capital Medical University; 3 Shijitan Hospital, Capital Medical University

**Keywords:** Opioid, immune function, lymphocytes, natural killer cells, macrophage

## Abstract

Opioid receptors and their ligands produce powerful analgesia that is effective in perioperative period and chronic pain managements accompanied with various side effects including respiratory depression, constipation and addiction etc. Opioids can also interfere with the immune system, not only participating in the function of the immune cells, but also modulating innate and acquired immune responses.

The traditional notion of opioids is immunosuppressive. Recent studies indicate that the role of opioid receptors on immune function is complicated, working through various different mechanisms. Different opioids or opioids administrations show various effects on the immune system: immunosuppressive, immunostimulatory, or dual effect. It is important to elucidate the relationship between opioids and immune function, since immune system plays critical role in various physiological and pathophysiological processes, including the inflammation, tumor growth and metastasis, drug abuse, and so on. This review article tends to have an overview of the recent work and perspectives on opioids and the immune function.

## Introduction

Analgesic drugs, especially opioids, have been a major focus of medical research due to their critical roles in pain management. Approximately one-third of the adults in the United States suffer from certain chronic pain annually, and more have acute pain associated with trauma or surgery. Opioids are typical central analgesics, which produce powerful analgesia that is effective in treating severe pain. Apart from their analgesic effects, opioids have been shown to affect multiple organs and systems including the immune system through various mechanisms.

Opioids interact with opioid receptors on the cell membrane and play an important role in physiological and pathophysiological process. The opioid receptors family consists of three classical receptors: μ, δ and κ opioid receptors, all of which belong to the G-protein coupled receptors (GPCR) family with seven transmembrane domains. They are expressed not only within the central nervous system but also on peripheral sensory nerve terminals. Opioid receptors have complex biological and pharmacological properties. Not only do they play important role in analgesia, drug tolerance, addiction, depression, and respiratory depression, but also in the cardiovascular and immune system.

Each of the three most classical types of receptors has their own endogenous ligands and unique functions. Endorphin, the endogenous ligand for μ receptors, has a significant impact on analgesia, respiratory inhibition, and the heart rate reduction. Although Enkephalin, a δ receptors’ endogenous ligand, has no significant analgesic effect, and is involved in the protection of myocardial ischemia. Dynorphin, the endogenous ligand for κ receptors, has analgesic properties and can induce anxiety with very weak respiratory inhibition effects.

Numerous studies have shown that there is a close connection between neuroendocrine and immune systems. There are many opioid receptors on a different kind of immune cells according to the previous research^1–5^. The nervous system may release opioid peptides that can combine with the opioid receptors on the membrane of immunocytes to regulate the immune function. Moreover, immunocytes can regulate the immune function by secreting opioid peptides that can adjust the neuroendocrine system. It is previously believed that most opioids suppress the immune system, but recent research indicates they may play a dual effect. However, the mechanism of how the opioids and opioid receptors work in the immune system is still not clearly understood. In this review, we will discuss the relationship between opioids and immune system.

## Effects of opioids on immune cells

### T lymphocytes

T lymphocytes are the primary cells of human cellular immunity, and they also regulate the activity of other lymphocytes, monocytes, and natural killer cells via neuroendocrine mechanism or cytokines. Dated back to 1979, Wybran’s research reported on the modulation of rosette formation of human T lymphocytes by opioids^1^. Since then numerous immunomodulatory effects of opioids on T lymphocytes have been reported and reviewed.

In 1988, Sibinga and Goldstein published the first review that addressed whether cells from the immune system express opioid receptors^6^. From then on, more and more evidences indicate that T lymphocyte express all three kinds of opioid receptors^7^.

μ receptors have been intensively studied in T cells. Morphine, a classic μ receptor agonist, can regulate various aspects of T lymphocytes functions. One of the effects of opioids is the immune modulation of the T helper cell balance. It is reported that some opioids can induce interleukin (IL)-4 to mediate partial anti-inflammatory effect. Fentanyl, methadone, loperamide and beta-endorphin induced a remarkable production of IL-4 on human T lymphocytes. On the contrary, morphine and buprenorphine resulted in a significantly lowered levels of IL-4 mRNA and protein^8^. This ligand-biased phenomenon may be due to different agonists at μ receptors in T cells induced different signaling pathways or activate certain signaling pathways to a significantly different extend. This should help to manage potential side effects of opioids more efficiently.

For the sake of identification of the effects of morphine on the immune response, most of the work has focused on analyses of acute effects of the drug. But opioids seem to show a different effect on the immune system by chronic administration. Researchers compared the peripheral blood samples of the patients who suffer chronic nonmalignant pain with healthy volunteers. After twelve months of treatment with intrathecal morphine in patient group, there was an increase in μ opioid receptor (MOR) mRNA levels in T lymphocytes (also in B lymphocytes). Even higher levels were observed in those patients treated with co-administration of morphine and bupivacaine. Elevation of MOR mRNA levels was confirmed in patients after twenty-four months of treatment. The mechanism is unclear, immune cells containing and releasing opioid peptides can accumulate in chronically inflamed tissue and act as cytokines to mediate morphine induced upregulation of MOR might be a reasonable explanation^9^. Cornwell first reported the effect of chronic morphine exposure on circulating T cell dynamics. By examining the effect of long-term morphine exposure on the circulating T cell population dynamics in rhesus macaques, he found that the numbers of circulating Treg cells and the functional activity of Th17 cells, were significantly increased with chronic morphine administration^10^. Whether the increased circulating Tregs and Th17 functionality by the chronic morphine administration is associate with the ultimate immune suppression need to be investigated.

Met—enkephalin, MENK), an endogenous ligands for δ receptors, alone or combined with IL-2 or IFN-γ, can up-regulate both CD_4_^+^ T cell expansion and the CD_4_ molecule expression in vivo and in vitro. Moreover it can increase the production of CD_4_^+^ T cells^11^. However, in contrast, some other studies found that the enkephalin helped the cancer cells to escape the host’s anticancer immunity through inhibits the expression of T lymphocytes, especially those belonging to the CD4^+^ subset in colorectal carcinoma patients^12^.

In recent years, the study of κ receptors increased. U50,488H, a κ receptors agonist, suppresses staphylococcal enterotoxin B induced proliferation of T lymphocytes and IL-2 production in vitro. Such effect can be reversed by nor-BIN and naloxone^13^. However, under in vivo conditions, U50,488H stimulated the proliferation of T lymphocytes, which was stimulated by lectin^14^. Although the result varies, data accumulated in investigations of the influence of κ receptors agonists on T lymphocytes indicate their significance in regulating T lymphocytes. Present findings indicate that signaling through different opioid receptor in T cells leads to different immune effects, the mechanisms of these actions by which cytokine production or signal pathway has not been clarified. The future studies should be conducted to investigate this issue.

### B lymphocytes

B lymphocytes are involved in the humoral immunity mainly by producing antibodies and memory cells. They also express μ, δ, and κ opioid receptors^7^. Some studies have showed that appropriate concentration of opioid substances like morphine may lead to the increase of IL-2, IL-6 secretion in T lymphocytes. IL-2 can stimulate B lymphocyte differentiation and enhance the function of producing antibodies, thus increasing the humoral immunity^2^. But implantation of a 75mg morphine slow-release pellet reduced the mitogenic responses of splenic B cells to bacterial lipopolysaccharide^15^ and this has been confirmed using an injection dosing regimen^16^.

Morphine-pellet reduces body temperature may be suggested to explain the reduction of mitogenic lymphocyte proliferation, or the suppression of these immunologic response may be secondary to the glucocorticoid mobilizing effect of morphine.

The μ and δ opioid receptor agonists induced IgM and IgG antibody production in peripheral blood and spleen was compared. μ opioid receptor agonist promoted an increase in the IgM and IgG immune reaction, whereas δ opioid receptor agonist showed the contrary effect^17^.

According to the literatures review, opioids do have influences on B cells. However, formation of an antibody response always requires interaction of macrophages, T cells, and B cells. Thus, depression of the capacity to produce antibody does not necessarily mean that the effect of the drug is on B cells, the question has been raised as to whether there is a direct effect of morphine on the cells involved in antibody formation, or if these effects are also mediated.

## Natural killer (NK) cells

NK cells are the specific cytotoxic lymphocytes and are the major components of the innate immune system. The effects of opioids on NK cells are ambiguous. The underlying mechanism of opioid-induced changes to human NK cells in vivo has not been well investigated.

Recent literatures suggest that the morphine-induced inhibition of NK cell activity does not relate to the opioid direct interaction with NK cells, but it may be as one consequence of the opioid receptor activation in the central nervous system. Regulation of NK cells activity seems to be related to the dose of opioids. High doses of morphine have shown inhibition capability in NK cell activity^3^. For instance, intrathecal administration of morphine significantly decreases in NK cell activity^18^. However, a low dose of morphine enhances NK cell cytotoxicity. In animal experiments, morphine induced the dose- and time-dependent changes in NK cells activity. Morphine in a dose of 0.5mg/kg induced 4-fold increase in NK cells activity. Higher morphine doses (1.0, 5.0 mg/kg) induced an early increase followed by a decrease in cytotoxicity^19^. Some authors believe that ligands of κ receptors are not related to the regulation of activity of the NK cell. Dynorphin A inject into the CNS in vivo had no influence on the activity of NK cells. Moreover, antibodies to dynorphin A have no influence on the functions of NK, whereas antibodies to β-endorphin inactivate the activity of these cells^20^. A human study using flow cytometry to analyze the CD56 surface expression intensities of NK cell in peripheral blood lymphocytes indicate that NK cells from opioid treated patients do not show any signs of NK cell immunesuppression^21^.

## Monocytes/Macrophages

In the innate immune system, macrophages are the first line of defense against invading pathogens. Their phagocytosis and chemotaxis play a significant role in the process of pathogens removal. Any dysfunction of macrophages is harmful to the host.

Morphine has been shown to alter a number of macrophage functions in humans, these include phagocytosis, tumoricidal activity, nitric oxide (NO) production, therefore, damaging the host’s ability to combat off invading pathogens^4^. But IFN-γ could strongly stimulate macrophages to express κ opioid receptors in both transcriptional and protein level. Just like IL-1β, IL-4, IL-6 and TNF-α, they all participate in up-regulation of μ opioid receptors on macrophage^22^.

In vitro studies have shown that opioids inhibit the Fcγ-receptor mediated phagocytosis. This kind of effect of opioids on phagocytosis by murine peritoneal macrophages is mediated by μ and δ-2 receptors, but not by κ opioid receptors^23^. In the typical acute inflammatory response, blood monocytes come into the infected tissue and differentiate into macrophages shortly after the recruitment of neutrophils. Recently there is a new mice model of long-term morphine treatment followed by respiratory infection with Diplococcus pneumonia to evaluate the relationship between opioid and immunocytes. Morphine treatment significantly delays the entry of neutrophils into the alveolar septum, leading to significantly fewer neutrophils in lung tissue and bronchial alveolar lavage fluid in the initial stage of infection^24^. According some reports, endogenous ligands have different effects on macrophages. β-endorphin and dynorphin can stimulate monocytes/macrophages release superoxide ion O_2_^−^ to participate in the oxidative disinfection processes, but Met-enkephalins or Leu-enkephalins inhibit such effect^25^.

An increasing number of studies indicate that there is a dose-dependent relationship between opioids and macrophages. One study has noted that the morphine plays a dose-dependent biphasic effect in mice infected with Leishmania. In 10^−11^–10^−9^M ranges, morphine can prevent Leishmania infection. Whereas in 10^−5^M ranges, it augments intramacrophage parasite growth^26^. The κ receptors agonist dynorphin A significantly and dose-dependently increases phagocytosis in peritoneal macrophages. The effect of dynorphin A on the phagocytic activity of the cells is stronger than effects of enkephalins, dynorphin B, and separate fragments of the dynorphin A molecule^25^. However, in the monkey model, the chemokine induced migration of monocytes and neutrophils in peripheral blood are suppressed by κ-opioid agonist U50,488H^27^. A substantial amount of research has been performed on the opioid immunomodulatory effects to monocyte/macrophages, but the results were diverse, the exact mechanisms need to be investigated.

## Dendritic cells

Dendritic cells are the most potent antigen-presenting cells that were discovered by Canadian scholar Steinman in 1973^28^. They play a critical role in the innate and adaptive immune system as important inducers and regulators in the immune response.

Makarenkova et al. have reported the expression of δ-and μ receptors on dendritic cells^29^. Activated dendritic cells can be regulated by endogenous opioid through its μ receptor. Receptors’ activation by endomorphin lead to IL-10 activation and IL-12 and IL-23 suppression^5^. In human bone marrow, δopioid receptor mRNA express at a low level in immature dendritic cells, conversely, at a high level in mature dendritic cells^30^. Some research have also found that MENK can significantly promote the maturation of dendritic cells and improve their functions. It moderates the relationship between dendritic cells and CD_4_^+^ T cells, and adjusts their immune function positively^31^. In addition, MENK at physiological concentrations exert positive immunoregulation on various types of immune cells, such as augmenting the interactions between dendritic cells and CD4^+^ Th1 cells, inducing phenotypic and functional differentiation/maturation of these cells with increased antigen presentation^32^. Some data also demonstrate that dendritic cells express functional κ opioid receptors and that specific agonists exert a direct effect on these cells. A fundamental aspect of dendritic cell function is their capacity to activate T cells. Selective κ-receptor agonists dynorphin A could directly modulate dendritic cells function through itsκreceptor, thus suppress the T cell proliferation by changing the regulation of the Th1-Th2 balance, but do not influence the antigen presenting function or the maturation processes^33^. These findings suggest that opioids from different classes might have the opposite effect on dendritic cells in respect to their ability to activate T cells. On dendritic cells, opioids showed a similar effect with the mediators of the neuroendocrine system, therefore, dendritic cells might be involved in the crosstalk between the nervous and immune system.

## Opioid receptors and inflammation

Inflammatory pain is primarily due to the activation of particular nociceptive neurons in the direct action of inflammatory mediators. Under this circumstance, there are two principal pharmacologic strategies of inflammatory pain in the periphery. First, the application of non-steroidal anti-inflammatory drugs inhibits cyclooxygenase-derived prostaglandin production, and as a result, decreases nociceptor sensitization. This effect eventually prevents the development of hyperalgesia in humans and animals. The second strategy is exemplified by some analgesic medications like opioids that can directly block constant nociceptor sensitization via peripheral actions^34^.

Immune cells can migrate to inflammatory tissue and affect the development of inflammation by different mechanisms.

A vicious cycle of inflammation, pain, and substance P release by morphine stimulation in mice cancer mode has been described. Mast cell is triggered by morphine treatment in the tumors. Activated mast cell degranulate and then release substance P and tryptase that may strengthen peripheral or intra-tumoral nerve activation leading to pain. Stimulated nerve fibers may trigger extra substance P releasing sequentially, thus increasing neuro-inflammation and driving a vicious cycle of mast cell activation, inflammation, disease development, and pain^35^.

Amid all the opioid receptors, κ receptors are of key interest as targets for new peripherally acting opioids because their activation is not associated with the peripheral side effects attributed to μ agonists. Two essential parameters of inflammation, plasma extravasation, and neutrophil migration, were used to evaluate the anti-inflammatory and antinociceptive effect of opioids. Local activation of κ opioid receptors decreased both two parameters of hyposiagonarthritis in a dose-dependent and antagonist-reversible manner in mice, in the company of its potent antinociceptive effect^36^. The current notion has suggested that activation of peripheral κ receptors directly inhibits inflammatory hyperalgesia through stimulation of the nNOS/NO signaling pathway. One result has shown that peripheral activation of κ receptors by partial agonist directly inhibits enduring inflammatory hyperalgesia induced by PGE_2_ in rodent, escaping central side effects. This effect seems to be dependent on activation of the κ receptors expressed by primary nociceptive neurons, triggering the phosphoinositide 3-kinase gamma (PI3Kg)/AKT, which is probably responsible for the stimulation of nNOS signaling pathway^34^.

Inflammatory bowel diseases (IBD) are chronic inflammatory ailments of the gastrointestinal tract with unclear etiology. One of the key features of this sort of disease is the up-regulation of opioid receptors in gastrointestinal tract tissues, both the mRNA and protein level^37^. The use of opioids, especially the μ receptors agonists, such as morphine for the medical treatment of inflammatory bowel disease is limited by central and peripheral side-effects. However, the application of κ receptors partial ligands, acting exclusively at peripheral sites, might become an encouraging alternative. Salvinorin A (SA) is the major active component of a Mexican plant Salvia Divinorum, whose effects are mediated mainly through peripheral κ receptors. Mouse models of colitis was treated with SA, and the effect was evaluated in four aspects, macroscopic score, myeloperoxidase activity, colon length changes and ulcer grade. The results indicate that SA may display anti-inflammatory activity in mouse models of experimental colitis. At meanwhile, it produces analgesia in acute intestinal inflammation^38^.

Opioid receptors have immunomodulatory effects, but nothing is known about their antiviral properties. Some researchers investigate the role of opioid receptors in viral respiratory tract inflammation. As an inspiring outcome, opioid receptors might offer powerful novel pharmacologic targets to ameliorate viral respiratory tract inflammation and prevent its long-term prognosis^39^.

## Opioid receptors and cancer immunity

The innate and adaptive immune systems provide vital protection against cancer. Cancer immunosurveillance involves immunocytes such as natural killer cells, lymphocytes. Opioids are the most effective analgesics and widely applied for the therapy of severe cancer pain. However, a considerable amount of studies has convincingly demonstrated that opioids, especially morphine and its derivatives, are immunosuppressive. This may cause unexpected side effects during pain management, particular in people suffering from cancer, whose immune system have already impaired. The administration of morphine alters immune status including suppression of natural killer cell activity, mitogen-induced T and B lymphocyte proliferation, antibody formation, and cytokine production.

Some patients were administered opioids over a period of several months and others were administered opioids in a short time, such as patients just underwent surgery. More and more studies showed the immunologic consequences of opioids are likely to be very different in patients with chronic cancer pain. In patients with metastatic cancer, no significant changes were found in the levels of any cytokine after eight days of treatment with morphine^40^. However, early studies showed that the synthesis and secretion of IL-2 by lymphocytes increased significantly after four weeks of morphine treatment in patients with similar pain condition^41^. This may have suggested that the acute effects of opioids on the immune system are different from chronic exposure. In advanced cancer patients, the impact of opioids on immune function correlated with the time of opioid administration. There was a negative correlation between the levels of morphine metabolites and the levels of circulating immunoglobulin in patients who had just been commenced on morphine. However, such effects were not observed in patients who had been administered with morphine for more than one month^42^. Some other researchers summarized the effect of κ-opioid receptor ligands in vascular development in tumors. It demonstrated that κ-opioid receptor ligands as anti-angiogenic factors, which impede the vascular development by inhibiting the expression of vascular endothelial growth factor (VEGF) receptors^43^. It is therefore supposed that the regulation of responses to κ-opioid receptor ligands, which could be useful for managing vascular formation under physiological or pathological condition, offering medical benefits beyond the relief of pain.

Increasing study revealed that opioid treatment might influence the cancer outcome. A mice cancer model was utilized to verify if morphine can affect tumor development. Discovery showed that morphine did not influence tumor development when given before the onset of tumor appearance, but it promoted the development of the established tumors with increased μ-opioid receptors expression and reduced animals’ survival^35^. What’s more, in a human study, μ-opioid receptors expression was increased significantly in cancer samples from patients with lung carcinoma compared with adjacent normal tissue. It then seemed that the high expression of μ-opioid receptors in tumor tissue was a direct evidence that the opioids can affect tumor progression and opioid antagonist might become a novel therapeutic option for tumor treatment^44^. In addition, after either in vivo or in vitro treatment with MENK, result significantly increased the survival of tumor-bearing mice and shrank the tumor growth. Scientists attributed this phenomenon to MENK treatment, which could up-regulate the percentage of CD8^+^ T cells and induced cytotoxic activity, thereby against tumor cells and increasing secretion of INF-γ^45^.

## Opioids and immune-related drug addiction and abuse

As early as 1986, scientists irradiated morphine addiction rats by a gamma ray and found that the ruin of the immune cells in rats could alter the opiate withdrawal phenomena. In that experiment, radiation, either prior to or after chronic morphine treatment, significantly reduced the opiate withdrawal syndrome by naloxone. Hence, it was estimated that that addiction to opiates was in part related to the immune system^46^. Drug abuse has generated an important public health issue and a controversial social problem. Heroin, morphine, and other opiate analgesics are still to be frequently misused in the world.

Opiate abusers are mostly suffered multiple organ failure. Impairment of immune function is part of the most severe and frequent complications. Either in vitro studies with innate immune cells from animal models and humans or in vivo studies in experimental animals, opiate abuse injured the innate immunity and is accountable for increased liability to bacterial infection^24, 47–49^.

Many investigators and clinical practitioners devote themselves to search the worthwhile novel therapeutic agents for decreasing the opioid adverse effect and preventing opioid addiction or tolerance. For managing heroin dependence, a methadone maintenance treatment (MMT) was introduced in the mid-1960s. It has been an effective method for treating heroin-dependent patients and it has been widely used for many years^50^. In Italy, a study was to investigate immune system function between heroin addicted patients who had received methadone or buprenorphine maintenance treatment for six months and untreated heroin addicts. There was an encouraging finding that immune system abnormalities in heroin addicted patients could be restored to almost normal values by rational treatment with methadone and buprenorphine^51^.

However, long-term MMT or higher methadone dosage might affect immune system functions, thereby exacerbating the consequences of systemic inflammation and causing subsequent neuronal inflammation and damage. In the group of long-term methadone-maintained patients, proinflammatory cytokines IL-1β, IL-6 and IL-8 were significantly higher than those of healthy people. The IL-1β was significantly correlated with the duration of methadone maintenance treatment, and plasma TNF-α and IL-6 were significantly correlated with the daily methadone dosage administered^52^.

## Conclusion

Although studies have demonstrated that opioids can have differential effects on the immune system and differential interaction within the immunocytes, the conclusions are variable. Not all opioid drugs share the same immune profile, some opioids seem to have no effects on immune function, whereas others tend to be immunosuppressive or immunostimulatory. This is probably due to a combination effect of opioid drugs’ direct effects on immunocytes, indirect effects in vivo that involved centrally mediated mechanisms and the systemic production and release of immunomodulatory mediators.

There are many examples showing that individual opioids can affect the immune system in different ways. Short term/ low dose administration of opioids seems to have a positive impact on the immune system. Comparatively, long term/ high dose administration has a negative impact. Without a doubt, it is apparent that the possibility to reach adequate and equivalent pain control choosing either proper opioid drugs or the suitable administration of opioid drugs could represent an important point to be considered in the future opioid therapy.

Moreover, opioids play a different role in inflammation, cancer process and addiction due to their different effect on the immune system. On one hand, they could prevent inflammation, inhibit tumor growth and ameliorate addiction. They could aggravate inflammatory reaction, help the tumor escape from the immune immunosurveillance, induce addiction and increase the rate of infection.

In summary, since opioids and opioid receptors were discovered, they have been one of life science research hotspot. To clarify the mechanisms of analgesia, tolerance, addiction of opioid, the relationship among opioid, inflammation and cancer are the main targets of investigations. The development of new suitable opiate drugs depends on the underlying mechanisms about opioid receptors and related proteins. Variable modulatory effects on the immune system, the nervous system, and the endocrine system have been presented. How to exert the immunomodulatory effect of opioid to develop optimum clinical therapies without intolerable side effects, which is the key point for translation of basic research to clinical practice.
